# Insecticidal activity of *Thymus pallescens* de Noë and *Cymbogon citratus* essential oils against *Sitophilus zeamais* and *Tribolium castaneum*

**DOI:** 10.1038/s41598-024-64757-3

**Published:** 2024-06-17

**Authors:** Dahou Moutassem, Tahar Boubellouta, Yuva Bellik, Zyed Rouis, Dmitry E. Kucher, Aleksandra O. Utkina, Olga D. Kucher, Olga A. Mironova, Nyasha J. Kavhiza, Nazih Y. Rebouh

**Affiliations:** 1 Laboratory of Characterization and Valorization of Natural Resources (L.C.V.R), Faculty of Life and Natural Sciences and of Earth and Universe Sciences, Mohamed El Bachir El Ibrahimi University, 34000 Bordj Bou Arreridj, Algeria; 2 Laboratory of Genetic, Biodiversity and Bio-Resources Valorization, Higher Institute of Biotechnology of Monastir, University of Monastir, Monastir, Tunisia; 3 Department of Environmental Management, Institute of Environmental Engineering, RUDN University, 6 Miklukho-Maklaya St., Moscow, Russia 117198; 4 V.V. Dokuchaev Soil Science Institute, Pyzhyovskiy Lane 7 building 2, Moscow, Russian Federation 119017

**Keywords:** *Cymbopogon citratus*, Energy biomarkers, *Sitophilus zeamais*, *Thymus pallescens* de Noé, *Tribolium castaneum*, Ecology, Plant sciences, Ecology, Environmental sciences, Chemistry

## Abstract

The thrust of the study was to determine the chemical composition of the essential oils extracted from *Thymus pallescens* de Noé and *Cymbogon citratus* Stapf. as well as to evaluate their efficacy in controlling *Sitophilus zeamais* Motschulsky and *Tribolium castaneum* (Herbst) in either single or combined populations. Carvacrol (56.04%) and geraniol (20.86%) were identified as the major constituents of *T. pallescens* and *C. citratus* respectively. The tested essential oils showed pronounced insecticidal activity against the pest species in relation with the applied doses. *T. pallescens* EO had the highest efficacy and *S. zeamais* was found to be more susceptible to both individual and combined treatments. With reference to the contact and fumigation assessments, *T. pallescens* EO effectuated corrected mortality rates ranging from 42.5–100% to 25–100% in *S. zeamais* with corresponding lethal concentration (LC_50_) values of 17.7 µl/ml and 15µL/L air respectively. Whereas, the *T. pallescens* EO exhibited corrected mortality rates of 42.5–100% and 20–100% with corresponding LC_50_ values of 18.1 µl/ml and 15.5 µL/L air against *T. castaneum* in contact and fumigation assessments, respectively. The corrected mortality rates increased for both insect species when using combination treatments, with significant increases in the LC_50_ values, ranging from 8.59 to 49.9% for both pest species. Analysis of energy biomarkers in the treated insects indicate significantly increased protein and carbohydrate contents and decreased lipids levels. The study therefore demonstrated the bio-insecticidal toxicity of the EOs from *T. pallescens* and *C. citratus* against two important maize post-harvest pests, concurrently revealing significant positive and negative insecticidal activity gradients in relation to single or combined populations.

## Introduction

Maize (*Zea mays* L.) is an important source of nutrients for both humans and livestock^1^. Maize production has increased significantly in recent years to keep up with the surging global demand^2^. However, several biotic and abiotic factors affect the productivity of maize, especially factors associated with post-production storage losses.

Pests are regarded as a principal cause of stored maize grain losses, estimated at 12–36% annually^3,4^. More than 37 species of insects have been reported to infest stored maize grains^5^. Notably, the major losses are mainly associated with Coleopteran species^3,4,6^.

The maize weevil *Sitophilus zeamais* Motschulsky (Coleoptera: Curculionidae) is considered as one of the most serious and damaging pests identified in stored corn worldwide. It affects the quantity and quality of the maize grains thus causing severe deterioration of the seed germination potential due to its ability to penetrate into the grain mass^4,7^. *S. zeamais* is a polyphagous pest species, which accounts for approximately 10–30% yield losses of the annual stored maize grains^8^.

Moreover, maize damaged by *S. zeamais* activity becomes highly susceptible to other saprophagous species especially *Tribolium castaneum* (Coleoptera: Tenebrionidae). In turn, infestation with *T. castaneum* is synonymous with increase in temperature and moisture of stored grains generating an environment that promotes fungal propagation leading to further grain degradation and deterioration^9^. Furthermore, co-existence of different species brings about interspecific competition which results in dramatic losses of the stored grain^10,11^.

To control insect pests during grain storage, chemical synthetic insecticides are frequently used since they can rapidly decimate dense insect populations. However, the indiscriminate use of these chemical compounds results in several adverse environmental impacts such as pest resistance, environmental pollution, disruption of ecological balance and destruction of non-target pollinator insects like bees^12,13^.

Indeed, resistance to several insecticides, such as malathion, pirimiphos-methyl, fenitrothion, and phosphine, has been reported in many storage insect-pests, including *S. zeamais* and *T. castaneum*^14,15^. Due to these adverse effects of synthetic chemical pesticides, biopesticides have been explored as a potential alternative^16,17^. In fact, natural insecticides have the advantage of low toxicity for humans and increased biodegradability^18^.

Several aromatic plants have been identified as potential organic pesticides that can control post-harvest insect pest species^19–23^. For example, *Thymus pallescens* essential oil finds extensive application in phytotherapy. Thyme is recognized for its antioxidative, antibacterial, insecticidal and anti-fungal properties^24–26^. More so, it has been documented that *Cymbopogon citratus* possesses various biological properties, encompassing insecticidal, antibacterial, antifungal and antioxidant activities^26–29^.

*S. zeamais* and *T. castaneum* have been found to be sensitive to various EOs and their active constituents^30,31^. Nonetheless, there is a scarcity of experimental work designed on the effects of EOs on simultaneous infestation of *S. zeamais* and *T. castaneum*. Therefore, the aim of this study was to determine the chemical compositions of *T. pallescens* and *C. citratus* EOs and to evaluate their insecticidal activity on 2 maize post-harvest pests, *S. zeamais* and *T. castaneum*, in single and combined populations. Consequently, we hypothesize that these EOs could negatively influence the physiological responses of the investigated pests.

## Material and methods

### Plant material collection and air curing

The aerial plant parts of *T. pallescens* and *C. citratus* were collected during the flowering season from various localities of Mascara and Algiers, North Algeria. All samples were partially dried for 15 days at room temperature (25 ± 3 °C).

### Insect rearing

The study focused on two Coleopteran species: *S. zeamais* and *T. castaneum*, which were sampled from infested seed maize from farmers’ maize stocks. The insects were reared separately in plastic jars (60 cm × 40 cm × 12 cm) containing a mixture of 1 kg commercial maize and commercial flour according to the proportion of 9/1 (w/w), respectively. The trays were maintained at 26 ± 3 °C and, 70 ± 10% relative humidity (RH), under a 12:12 h (L;D) photoperiod. The adult insects used in all toxicity and biochemical studies were 2-week post-emergence insects of mixed sex^13^.

### Essential oil extraction and GC–MS analysis

All EOs were obtained from the aerial parts of *T. pallescens* and *C. citratus* by hydrodistillation process for 3 h, using a Clevenger-type apparatus. Initially, a quantity of 100 g of plant material was added to 800 ml of distilled water in a 2-L flask. The set was placed in a balloon heater attached to a refrigerator to ensure condensation of EOs. The yields of EOs were expressed in g relative to 100 g of dry vegetable matter^32^. The *T. pallescens* leaves gave the highest yield of Eos, ranging from 1.8 to 2.1 g/100 g. The yield of *C. citratus* ranged from 0.8 to 1.1 g/100 g (mean value for four months). The extracted EOs were dried over anhydrous sodium sulfate and stored at 4°C until use.

Chemical analyses were performed using a system comprised of a 6890 gas chromatography (GC) apparatus using a VF WAX and HP-5MS capillary column (60 m × 0.25 mm × 0.5 µm film thickness) coupled with a Hewlett-Packard computerized 5973A mass spectrometry (MS) apparatus. All EOs were diluted 1/10 (v/v) in hexane, and 1 µL was injected by splitting at a ratio of 1:25. The GC column was a 30m (60m × 0.25mm × 0.5µm film thickness) using a VF WAX and HP-5 capillary column. The GC conditions were as follows: injector temperature, 250 °C; column temperature, isothermal at 60 °C and held for 6 min, then programmed to 250 °C at 6 °C/min and held at this temperature for 2 min; ion source temperature, 250 °C; and detector temperature, 320 °C. Helium was used as the carrier gas at a rate of 0.5 mL/min. The mass range varied from *m*/*z* 30 to 350 amu (atomic mass units). The EO components were identified by comparing their retention indices and mass spectra results against those for authentic samples and comparisons of the linear retention indices against a series of n-hydrocarbons. Computer matching was performed against both commercial (NIST 98 and ADAMS) and home-made library mass spectra, based on the analysis of pure substances and the components of known oils derived from data in the MS literature^33^.

### Insecticidal activity

The insecticidal activity of the EOs was evaluated against two pest species using two approaches: a separate treatment (each species was treated individually) and a combined treatment (the two species in coexistence). All bioassays were performed under controlled conditions (26 ± 1 °C, 65 ± 5% RH, and a 12:12 h L:D photoperiod).

#### Contact toxicity

Contact toxicity assays were conducted in accordance with a previously described protocol^34^. Each EO was dissolved in acetone to prepare a serial dilution. Preliminary tests were run to determine the appropriate concentrations that can cause mortality rates ranging from 20 to 80% mortality. For both species, concentrations of 1, 5, 10, 20, 30, 40, 50, 100, 200, 400 µL/mL of each EO were tested. Guided by this assessment the following concentrations were selected for the study: 10, 20, 30, 40, and 50µL/mL. The EOs were applied on the filter paper discs (9 cm diameter) using 2 mL for each trial. Acetone was allowed to evaporate for 10 min prior to the introduction of pest species. Twenty unsexed adults of *S. zeamais* and *T. castaneum* were introduced, either separately or together, into Petri dishes containing treated filter paper. The control was maintained under the same conditions without EOs. Four replicates were performed for each treatment. The mortality rates were recorded 72 h after treatment and corrected using Abbott’s formula (1925). The insects were considered dead when no movement was recorded. Half-maximal lethal concentration (LC_50_) values were assessed using probit analysis.

#### Fumigant toxicity

The fumigant toxicity of EOs was evaluated against *S. zeamais* and *T. castaneum* adults in accordance with a previously described technique^13^. Glass jars (1 L) were used as fumigation chambers. As for contact toxicity assay, preliminary tests were conducted in order to determine the concentrations that can effectuate 20 to 80% mortality rates in each of the insect populations. The tested concentrations were 1, 5, 10, 20, 30, 40, 50, 100, 200, 400 µL/L air. Subsequently the following concentrations of EOs were selected and used in the study: 10, 20, 30, 40, and 50 µL/L air. Filter papers (Whatman No. 1) were cut into 9 mm diameter pieces and sprayed with EOs then strongly affixed onto the undersides of the screw caps of the jars. The insides of the jars were brushed with Vaseline to prevent direct contact between the insects and the EO. Caps containing the treated filter paper were tightly screwed onto jars containing twenty adults of *S. zeamais* and *T. castaneum* either individually or together. The cover was well-sealed with parafilm. Control insects were maintained under the same conditions with affixed filter paper without EOs. The treatments were replicated four times. Mortality rates were recorded after 72 h of treatment and corrected using Abbott’s formula (1925). The LC_50_ values were assessed by probit analysis.

### Effect of *EOs* on energy biomarkers, including proteins, carbohydrates, and lipids

To determine proteins, lipids, and total carbohydrate contents, adult insects were treated with three concentrations (30, 40, and 50 µL/mL) of *T. pallescens* and *C. citratus* EOs. The control insects used in these experiments were insects that suffered natural deaths. Four replicates were performed for each analysis.

#### Effect of *EOs* on proteins

A technique described by Bradford^35^ was used for the extraction and quantification of protein reserves. Coomassie Brilliant Blue G-250 (100 mg) was dissolved in 50 mL 95% ethanol (Prolabo; 96% Purity), and 100 mL 85% (w/v) phosphoric acid (Sigma) was added. The resulting solution was diluted to a final volume of 1 L. After crushing the individual insects in 400 µL of the Tris–HCl (20mM) (Sigma; Purity:99.5%) solution, the samples were incubated at 4 °C for 30 min to allow the proteins time to dissolve. An aliquot of 0.1 mL was transferred to a 12 × 100-mm test tube, 5 mL of Bradford reactive was added to the test tube and the contents were mixed by vortexing. The protein concentration was determined by spectrophotometry at 595 nm. The protein concentrations of each sample were determined against a standard curve constructed using 125, 250, 500, 1000 and 2000 µg bovine immunoglobulin G (IgG) dissolved in the same buffer as the samples. Before reading, the plates were gently stirred for 5 s to separate the protein aggregates.

#### Effect of *EOs* on carbohydrates

For the extraction and quantification of total carbohydrates, previously described methods were used^36,37^. Insects were ground in 400 µL of sodium sulfate (Prolabo) for 2 min, followed by the addition of 2 mL methanol (Prolabo; 99.8 purity). The tubes containing the homogenate were then centrifuged at 4 °C for 4 min at 2000 × *g* for 2 min. An aliquot of 100 µL was transferred to a new 12 × 75-mm tube, and 2 mL anthorone reactive was added followed by incubation in a 95 °C water bath for 17 min. The tubes were then placed in an ice bath for 10 min and the optical density at 625 nm was measured. For carbohydrates, a calibration standard curve was generated using a standard glucose solution (1 g/L). The blank was a 0.5 mg/mL glucose solution (5 mg of glucose in 10 mL of distilled water). A series of dilutions were performed to obtain the following glucose concentrations: 10, 20, 40, 60, 80, 100, and 200 µg/mL.

#### Effect of *EOs* on lipids

Lipid content was determined using methods described by Van Handel^38^ and Plaistow et al.^39^. The insects were crushed in 400 µL chloroform/methanol (1:1, v:v) solution. The supernatant was transferred to a clean tube (16 × 100 mm), which was retained in a water bath at 95 °C, placed inside a fume cupboard to allow the remaining solvent to evaporate. Then, 200 µL of concentrated (95%) sulfuric acid was added, and the solvent was allowed to evaporate at 90 °C for approximately 10 min. The sample was removed from the heating bath, allowed to cool, and 5 mL vanillin-phosphoric acid reagent (85%) was added. The samples were vortexed and then exposed to open air for 5 min to allow coloration to develop. The optical density of each sample was measured at 525 nm read after 25 min. The lipid concentration for each sample was determined against a standard curve constructed using 25, 50, 100, 200, 400, 800, and 1200 µg of commercial vegetable oil.

### Data analysis

Generalized linear models (GLMs) were used to analyze the corrected mortality (CM%) and energy biomarker values. The CM% was subjected to probit analysis to obtain LC_50_ values with their confidence limits. The insect corrected mortality and energy biomarkers data for contact and fumigant tests were separately subjected to Multivariate Analysis of Variance (MANOVA) with pest species, essential oil type, insect occurrence (single or combined) and concentrations of EOs as main effects. Mortality was the response variable. Mean comparison was performed using Tukey’s post hoc test at 5% probability level. All analyses were performed using the statistical software R Studio 1.2.5019- R version 3.6.1.

## Results

### Chemical composition of *EOs*

Chemical compositions of *T. pallescens* and *C. citratus* EOs are given in Table 1. The chemical analysis of *T. pallescens* EO revealed more than 18 compounds, representing 92.38% of the total EO, and the major constituents were carvacrol (56.64%), p-cymene (16.36%) and thymol (8.71%). The chemical profile of *C. citratus* EO revealed more than 30 compounds, comprising 82.75% of the total EO components, with geraniol (20.86%), limonene (10.5%), and camphene (7.8%) identified as the major constituents.

**Table 1:** Chemical constituents of the essential oil from *T. pallescens* and *C. citratus* as analyzed by GC–MS.

Number	Compounds	RT (min)	Composition (%)	
			*T. pallescens*	*C. citratus*
1	α- Thujene	5.68	0.35	–
**2**	**α-Pinene**	**5.85**	**3.33**	**2.18**
**3**	**Camphene**	**6.2**	**0.30**	**7.80**
4	α-Pinene	6.84	0.36	–
5	α -Terpinene	7.79	1.15	–
6	β -Myrcene	7.11	0.65	0.5
**7**	**Limonene**	**13.8**	**0.73**	**10.5**
8	1.8-Cineole	14.2	0.34	t
9	β -Phellandrene	14.4	–	0.31
**10**	**γ -Terpinene**	**16.3**	**5.40**	**t**
**11**	**p-Cymene**	**17.6**	**16.36**	**0.12**
12	α -Terpinolene	18.2	0.19	0.4
13	α -Gurjunene	33.5	t	–
**14**	**Citronellal**	**30.3**	–	**4.37**
**15**	**Linalol**	**34.6**	**3.12**	**0.70**
16	Bornyl acetate	36.5	–	0.69
17	β -Elemene	37.2	–	1.07
18	β-Caryophyllene	37.5	–	1.02
19	Terpinene-4-OL	37.8	0.33	–
20	Citronellyl acetate	41.6	–	0.89
21	α -Terpineol	43.6	–	2.19
22	Terpenyl acetate	43.7	–	0.23
**23**	**Borneol**	**43.8**	**0.76**	**4.72**
24	Germacrene D	44.5	–	0.87
25	Geranial	45.6	–	0.92
26	Bicyclogermacrene	45.7	–	1.6
27	α -Muurolene	46.5	–	0.43
**28**	**δ -Cadinene**	**47.2**	–	**4.18**
**29**	**Geranyl acetate**	**47.3**	–	**3.39**
30	Citronellol	47.7	–	2.81
**31**	**Geraniol**	**52.4**	–	**20.86**
32	Caryophyllene	59.5	0.57	0.32
33	Caryophyllene oxyde	56.01	0.30	–
**34**	**Methylisoeugenol**	**60.9**	–	**7.1**
35	Elemol	64.6	–	1.37
**36**	**Thymol**	**69.6**	**8.71**	–
37	α -Eudesmol	71.2	–	0.34
**38**	**Carvacrol**	**71.7**	**56.64**	**t**
39	β -Eudesmol	71.8	–	0.32
40	α -Cadinol	71.9	–	0.37
	Total (%)		92.38	82.57

*RT* retention time; trace.

Significant values are in bold.

### Insecticidal activity

The tested EOs exhibited strong insecticidal activity against *S. zeamais* and *T. castaneum* adults. A behavioral change was also noticed in both treated insect pests, as indicated by the observation of increased aggregation and couplings between males and females.

#### Contact toxicity

During contact toxicity bioassay, both *T. pallescens* and *C. citratus* EOs showed strong insecticidal activity towards *S. zeamais* and *T. castaneum* adults (Table 2). The intensity of CM% varied according to the target pest (*F* = 70.1*; P* = 0.000*), the type of EO (*F* = 31.6; *P* = 0.000*), the applied concentration (*F* = 138.2; *P* = 0.000*), and whether the species were alone or combined (*F* = 7; *P* = 0.09*). It was found that both insect pests were more sensitive to *T. pallescens* EO compared to *C. citratus* EO. The CM% gradually increased with increasing EO concentrations. Under separate population treatments, *T. pallescens* EO (10–50 µL/mL) resulted in CM% values of 35.0–100% and 42.5–90% for *S. zeamais* and *T. castaneum*, respectively, whereas CM% values for *C. citratus* EO (10–50 µL/mL) were 12.5–65% and 5–42.5%, respectively. When the contact tests were carried out on combined populations (*S. zeamais* and *T. castaneum*), the results revealed a significant adulticidal activity of EOs against these insect species. At the same application rates and the same exposure period, the CM% values for *T. pallescens* and *C. citratus* EOs (10–50 µL/mL) on *S. zeamais* adults were 10–100% and 5–77.5%, respectively, whereas the CM% values for *T. castaneum* adults were 15–80% and 5–35%, respectively.

**Table 2:** Effect of contact toxicity of *T. pallescens* and *C. citratus* EOs against *S. zeamais* and *T. castaneum* adults in single and combined populations.

Occurrence mode	Dose (µL/mL)	*T. pallescens*		*C. citratus*	
		*S. zeamais*	*T. castaneum*	*S. zeamais*	*T. castaneum*
Single	10	42.5 ± 1.4^bc^	42.5 ± 8.5^cde^	12.5 ± 1.4^cd^	05.00 ± 2.8^b^
	20	35.0 ± 2.9^cd^	52.5 ± 9.5^bcd^	16.2 ± 3.7^cd^	07.50 ± 4.7^b^
	30	57.5 ± 2.5^bc^	52.5 ± 4.7^bcd^	16.2 ± 2.9^cd^	22.5 ± 2.5^ab^
	40	100. ± 0.0^a^	75.0 ± 9.5^ab^	35.0 ± 6.4^bc^	32.5 ± 4.7^a^
	50	100. ± 0.0^a^	90.0 ± 4.0^a^	65.0 ± 2.8^a^	42.5 ± 8.6^a^
Combined	10	10.0 ± 4.0^d^	15.0 ± 2.8^e^	5.0 ± 0.0^d^	05.0 ± 2.8^b^
	20	50.0 ± 7.0^bc^	15.6 ± 6.4^e^	37.5 ± 11.0^bc^	07.5 ± 2.5^b^
	30	65.0 ± 13.2^b^	27.5 ± 2.5^de^	25.0 ± 6.4^cd^	10.0 ± 4.0^b^
	40	95.0 ± 5.0^a^	60.0 ± 9.1^abc^	57.5 ± 4.7^ab^	32.5 ± 4.7^a^
	50	100. ± 0.0^a^	80.0 ± 4.0^ab^	77.5 ± 4.7^a^	35.0 ± 8.6^a^
Control		0.00. ± 0.0	0.00. ± 0.0	0.00. ± 0.0	5.00. ± 0.0

Values represent the mean of four replicates ± SE (standard errors). Data marked by different letters in a column indicate significant difference at P < 0.05.

The results of the contact toxicity trial expressed as LC_50_ of *S. zeamais* and *T. castaneum* adults, when tested separately or in combination are shown in Table 3. In the separate population treatments*, T. pallescens* EO was more active on *S. zeamais* (LC_50_ = 17.7 µL/mL) and *T. castaneum* adults (LC_50_ = 18.1 µL/mL). *C. citratus* EO was more effective against *S. zeamais* adults than against *T. castaneum* with LC_50_ values of 46.2 and 57.4 µL/mL, respectively. The LC_50_ values increased significantly under combined population treatment with levels of 8.59–49.9% for both insect pests. Similarly, *T. pallescens* EO demonstrated the highest insecticidal activity against *S. zeamais* and *T. castaneum* in the combined population with LC_50_ values of 20.8 and 36.1 µL/mL, respectively.

**Table 3:** LC_50_ values of contact toxicity of *T. pallescens* and *C. citratus* EOs against *S. zeamais* and *T. castaneum* adults in single and combined populations.

Pest species	Occurrence mode	EOs	LC_50_ (µL/mL)	Confidence interval (95%) (µL/mL)	Slope ± SE	${\left({\chi }^{2}\right)}^{\text{d}}$
*S. zeamais*	Single population	*C. citratus*	46.2 ± 2.8	40.3–52.1	1.1 ± 0.18	2.2
		*T. pallescens*	17.7 ± 1.4	14.7–20.6	1.9 ± 0.23	2.6
	Combined population	*C. citratus*	34.8 ± 2.8	28.9–40.8	1.7 ± 0.24	3.3
		*T. pallescens*	20.8 ± 1.6	17.4–24.2	2.2 ± 0.24	2.9
*T. castaneum*	Single population	*C. citratus*	57.4 ± 4.3	48.2–66.6	1.0 ± 0.11	1.3
		*T. pallescens*	18.1 ± 2.7	12.3–23.9	1.8 ± 0.19	3.6
	Combined population	*C. citratus*	62.8 ± 8.8	14.7–20.6	0.8 ± 0.16	2.0
		*T. pallescens*	36.1 ± 1.6	32.7–39.6	1.7 ± 0.21	2.5

Values represent the mean of four replicates ± SE (standard errors). *LC* median lethal concentration, *CL* confidence intervals.

#### Fumigant toxicity

The CM% values determined from the fumigant toxicity test clearly indicated that both populations of insect pest species were greatly affected following their exposure to the tested EOs (Table 4). The intensity of CM% varied according to the target pest (*F* = 7.9*; P* = 0.006*), the type of EO (*F* = 110.7; *P* = 0.000*), the applied concentration (*F* = 204.9; *P* = 0.000*), and whether the species were alone or combined (*F* = 8.7; *P* = 0.04*).

**Table 4:** Effect of fumigant toxicity of *T.pallescens* and *C.citratus* EOs against *S. zeamais* and *T. castaneum* adults in single and combined populations.

Occurrence mode	Dose (µL/L air)	*T. pallescens*		*C. citratus*	
		*S. zeamais*	*T. castaneum*	*S. zeamais*	*T. castaneum*
Single	10	25.0 ± 2.8^c^	20.0 ± 0.0^b^	20.0 ± 4.0^e^	17.5 ± 4.7^c^
	20	62.5 ± 2.5^b^	23.7 ± 2.3^b^	57.5 ± 10.3^c^	32.5 ± 4.7^bc^
	30	87.5 ± 2.5^ab^	31.5 ± 6.5^b^	75.0 ± 10.4^ab^	50.0 ± 4.0^b^
	40	100. ± 0.0^a^	35.0 ± 6.1^b^	92.5 ± 2.5^a^	80.0 ± 4.0^a^
	50	100. ± 0.0^a^	95.0 ± 5.0^a^	100.0 ± 0.0^a^	90.0 ± 5.7^a^
Combined	10	17.5 ± 4.7^c^	27.5 ± 4.7^b^	15.0 ± 2.8^e^	22.5 ± 2.5^c^
	20	62.5 ± 14.9^b^	27.5 ± 4.7^b^	27.5 ± 4.7^de^	27.5 ± 4.7^bc^
	30	77.5 ± 16.0^ab^	32.5 ± 2.5^b^	47.5 ± 2.5^cd^	32.5 ± 7.5^bc^
	40	100 ± 0.0^a^	75.0 ± 2.8^a^	77.5 ± 4.7^ab^	47.5 ± 2.5^b^
	50	100 ± 0.0^a^	92.5 ± 4.7^a^	85.0 ± 5.0^a^	95.0 ± .5.0^a^
Control		0.00. ± 0.0	0.00. ± 0.0	0.00. ± 0.0	05.00. ± 0.0

Values represent the mean of four replicates ± SE (standard errors). Data marked by different letters in a column indicate significant difference at P < 0.05.

Interestingly, the results of fumigant toxicity evaluation showed a pronounced increase in CM% compared with those of contact toxicity test. According to the GLM analysis, the population of *S. zeamais* appeared to have higher sensitivity to both EOs as compared to *T. castaneum*. The treatment of both separate and combined populations with *T. pallescens* EO showed higher CM% especially on *S. zeamais* which was more susceptible than *T. castaneum*.

Moreover, both pest species were more susceptible when treated alone than when treated together for both studied EOs. In the separate population, *T. pallescens* EO was found to be more active, with LC_50_ values of 15 and 15.5 µL/L air against *S. zeamais* and *T. castaneum*, respectively (Table 5). Under separate population treatments, *T. pallescens* EO (10–50 µL/mL) caused in CM% values of 25.5–100% and 20–95% for *S. zeamais* and *T. castaneum*, respectively, whereas CM% values for *C. citratus* EO (10–50 µL/mL) were 20–100% and 17.5–90%. A clear increase in the LC_50_ values (11.76 to 44.83%) was observed during the treatments of the combined population compared with the values measured in the separate population. *Sitophilus zeamais* was more sensitive than *T. castaneum* to the toxic effects of *T. pallescens* and *C. citratus* EOs, with LC_50_ values of 17 and 29.6µL/L air, respectively. When using contact test on combined populations (*S. zeamais* and *T. castaneum*), the CM% values for *T. pallescens* and *C. citratus* EOs (10–50 µL/mL) on *S. zeamais* adults were 17.5–100% and 15–85% for, whereas the CM% values for *T. castaneum* adults were 27.5–92.5% and 22.5–95%, respectively.

**Table 5:** LC_50_ values of fumigant toxicity of *T. pallescens* and *C. citratus* EOs against *S. zeamais* and *T. castaneum* adults in single and combined populations.

Pest species	Occurrence mode	EOs	LC_50_ (µL/L air)	CL (95%) (µL/L air)	Slope ± SE	${\left({\chi }^{2}\right)}^{d}$
*S. zeamais*	Single population	*C. citratus*	36.7 ± 3.1	29.9–43.4	1.6 ± 0.29	3.9
		*T. pallescens*	15.0 ± 3.7	7.0–22.9	0.9 ± 0.28	3.7
	Combined population	*C. citratus*	29.6 ± 2.9	23.5–35.8	1.7 ± 0.21	3.3
		*T. pallescens*	17.0 ± 1.8	13.1–20.9	2.0 ± 0.33	3.7
*T. castaneum*	Single population	*C. citratus*	26.0 ± 1.5	22.7–29.3	1.9 ± 0.15	2.2
		*T. pallescens*	15.5 ± 0.5	14.4–16.6	1.8 ± 0.18	1.1
	Combined population	*C. citratus*	62.8 ± 8.8	14.7–20.6	0.8 ± 0.16	2.0
		*T. pallescens*	36.1 ± 1.6	32.7–39.6	1.7 ± 0.21	2.5

Values represent the mean of four replicates ± SE (standard errors). *LC* median lethal concentration, *CL* confidence intervals.

### Effect of *EOs* on energy biomarkers

Protein content, lipid content, and carbohydrate levels were determined in *S. zeamais* and *T. castaneum* treated with *T. pallescens* as well as *C. citratus* EOs, and the results are shown in Tables 6 and 7.

**Table 6:** Effect of contact toxicity of *T. pallescens* and *C. citratus* EOs on protein, lipids, and carbohydrates quantities in *S. zeamais* and *T. castaneum* adults.

Pest species		*S. zeamais*			*T. castaneum*		
	EOs (µL/mL)	30	40	50	30	40	50
Proteins (µg/mg)	*T. pallescens*	13.25 ± 0.82^b^	14.83 ± 0.99^b^	15.81 ± 0.79^b^	12.39 ± 1.01^b^	13.34 ± 0.97^b^	15.27 ± 0.88^ab^
	*C. citratus*	12.57 ± 0.51^b^	13.91 ± 0.78^b^	15.81 ± 0.59^b^	12.12 ± 0.60^b^	13.86 ± 1.08^b^	15.71 ± 0.94^ab^
	*Control*	22.60 ± 1.41^a^	22.60 ± 1.41^a^	22.60 ± 1.41^a^	18.95 ± 0.12^a^	18.95 ± 0.12^a^	18.95 ± 0.12^a^
Lipids (µg/mg)	*T. pallescens*	1.60 ± 0.12^de^	2.88 ± 0.10^a^	2.95 ± 0.16_a_	2.25 ± 0.21^a^	2.24 ± 0.16^a^	1.95 ± 0.03^bc^
	*C. citratus*	1.52 ± 0.17^b^	1.86 ± 0.24^b^	2.02 ± 0.33^b^	1.83 ± 0.27^bc^	1.74 ± 0.12^bc^	1.73 ± 0.05^bc^
	*Control*	1.55 ± 0.16^b^	1.55 ± 0.16^b^	1.55 ± 0.16^b^	1.35 ± 0.20^c^	1.35 ± 0.20^c^	1.35 ± 0.20^c^
Carbohydrates (µg/mg)	*T. pallescens*	1.26 ± 0.09^b^	1.30 ± 0.02^b^	1.51 ± 0.04^a^	1.25 ± 0.08^b^	1.43 ± 0.02^b^	1.11 ± 0.05^b^
	*C. citratus*	1.09 ± 0.08^b^	1.17 ± 0.04^b^	1.18 ± 0.05^b^	1.43 ± 0.03^b^	1.42 ± 0.04^b^	1.00 ± 0.06^b^
	Control	1.93 ± 0.20^a^	1.93 ± 0.20^a^	1.93 ± 0.20^a^	2.00 ± 0.15^a^	2.00 ± 0.15^a^	2.00 ± 0.15^a^

Values represent the mean of four replicates ± SE (standard errors). Data marked by different letters in a column indicate significant difference at P < 0.05.

**Table 7:** Effect of fumigant toxicity of *T. pallescens* and *C. citratus* EOs on protein, lipids, and carbohydrates quantities in *S. zeamais* and *T. castaneum* adults.

Pest species		*S. zeamais*			*T. castaneum*		
	Eos (µL/L air)	30	40	50	30	40	50
Proteins (µg/mg)	*T. pallescens*	15.54 ± 0.08^c^	17.09 ± 0.33^b^c	19.87 ± 0.14^ab^	11.55 ± 1.08^c^	15.28 ± 0.69^abc^	16.74 ± 1.02^abc^
	*C. citratus*	16.75 ± 0.07^bc^	16.99 ± 0.73^bc^	22.86 ± 1.32a	14.80 ± 1.10^bc^	15.71 ± 1.44^abc^	18.42 ± 0.71^a^
	*Control*	22.60 ± 1.41^a^	22.60 ± 1.41^a^	22.60 ± 1.41^a^	18.95 ± 0.12^a^	18.95 ± 0.12^a^	18.95 ± 0.12^a^
Lipids (µg/mg)	*T. pallescens*	1.44 ± 0.03^b^	2.21 ± 0.19^a^	2.59 ± 0.12^a^	2.73 ± 0.18^a^	1.95 ± 0.13^b^	1.57 ± 0.13^bc^
	*C. citratus*	1.20 ± 0.04^b^	1.53 ± 0.18^b^	2.39 ± 0.19^a^	0.95 ± 0.13^d^	1.07 ± 0.04^d^	1.01 ± 0.01^d^
	*Control*	1.55 ± 0.16^b^	1.55 ± 0.16^b^	1.55 ± 0.16^b^	1.35 ± 0.20^cd^	1.35 ± 0.20^cd^	1.35 ± 0.20^cd^
Carbohydrates (µg/mg)	*T. pallescens*	1.37 ± 0.03^ab^	1.51 ± 0.08^ab^	1.68 ± 0.07^ab^	1.18 ± 0.07^c^	1.12 ± 0.05^c^	1.73 ± 0.13^ab^
	*C. citratus*	1.29 ± 0.06^ac^	1.36 ± 0.03^ab^	1.36 ± 0.07^ab^	1.32 ± 0.06^bc^	1.29 ± 0.06^bc^	1.01 ± 0.07^c^
	*Control*	1.93 ± 0.20^a^	1.93 ± 0.20^a^	1.93 ± 0.20^a^	2.00 ± 0.15^a^	2.00 ± 0.15^a^	2.00 ± 0.15^a^

Values represent the mean of four replicates ± SE (standard errors). Data marked by different letters in a column indicate significant difference at P < 0.05.

Protein content values were significantly affected by the type (*F* = 98.54; *P* = 0.000**) and the concentration of the used EO (*F* = 20.09; *P* = 0.000*), the pest species (*F* = 7.08; *P* = 0.000*), and the toxicity test type (*F* = 22.28; *P* = 0.000*). In contact toxicity, *S. zeamais* and *T. castaneum* treated with both EOs showed protein levels of 12.57–15.81 µg/mg and 12.12–15.71 µg/mg, respectively. Whereas, in fumigant toxicity *S. zeamais* and *T. castaneum* treated with both EOs presented protein levels of 15.54–22.86 µg/mg and 11.55–18.42 µg/mg, respectively. Overall, all tested EO concentrations reduced the protein content values in both pest insect species when compared to the control. Significant decreases in protein contents (18.47 to 48.87%) were observed for *S. zeamais* treated with *T. pallescens* and *C. citratus* EOs during contact and fumigant tests. The protein levels in *T. castaneum* under the same conditions decreased by approximately 9.77 to 33.67%.

The carbohydrate contents in *S. zeamais* and *T. castaneum* were significantly affected by treatment with *T. pallescens* and *C. citratus* EOs (*F* = 137.8; *P* = 0.0000*) but were not affected by pest species (*F* = 0.31; *P* = 0.56), the type of toxicity test (*F* = 0.11; *P* = 0.1), or the EO concentrations (*F* = 0.3; *P* = 0.73). In contact toxicity assay, the carbohydrate levels of *S. zeamais* and *T. castaneum* treated with both EOs were 1.09–1.51 and 1.0–1.43 µg/mg, respectively. The values recorded during fumigation were 1.29–1.68 and 1.01–1.73 µg/mg, respectively. These results were significantly lower than control levels. Carbohydrates contents were significantly decreased in *S. zeamais* (12.78–43.32%) and *T. castaneum* (13.34–49.33%) following during fumigant and contact toxicity tests using both EO treatments.

Lipid content values were significantly affected by the type (*F* = 102.1; *P* = 0.0000*) and concentration of EO (*F* = 36.1; *P* = 0.000*), the pest species (*F* = 27.5; *P* = 0.009*), and the type of toxicity test (*F* = 23; *P* = 0.000*). In contact toxicity, the highest levels of lipids were recorded in *S. zeamais* treated with *T. pallescens* (1.60–2.59 µg/mg) and *C. citratus* (1.52–2.02 µg/mg_)_ EOs. While, in fumigant toxicity the highest levels of lipids were recorded in *S. zeamais* treated with *T. pallescens* (1.44–2.95 µg/mg) and *C. citratus* (1.20–2.39 µg/mg_)_ EOs. This response was more pronounced with *T. pallescens* EO than *C. citratus* EO. The obtained results indicated a significant increase in the lipids contents of *S. zeamais* and *T. castaneum* treated with both EOs, with values ranging from 3.56 to 37.68% and from 2.8 to 52.81%, respectively.

## Discussion

In the conducted investigations, the chemical composition as well as insecticidal activity of *T. pallescens* and *C. citratus* EOs against *S. zeamais* and *T. castaneum* adults were assessed in separate and combined populations. According to the results, the major components of *T. pallescens* were identified as carvacrol (56.64%), p-cymene (16.36%), and thymol (8.71%). Our previous study revealed that carvacrol, thymol, γ-terpinene, and *p*-cymene were the major components of *T. pallescens* EO with percentage yields of 54.09, 16.24, and 8.47%, respectively, which is in quite agreement to the obtained results in this study^40^. The EO of the same plant (*T. pallescens*) has been reported to have similar composition^41^. Regarding *C. citratus* EO, geraniol (20.86%), limonene (10.50%), and camphene (7.80%) were found to be the major compounds, which is in accordance with previous findings^40,42^. However, the chemical composition of the *C. citratus* EO was different to that reported by Kumar et al.^43^ and Brügger et al.^44^ where neral, citral, 1,8-cineole, and geranyl acetate was detected at higher concentrations. These differences are probably due to the crop season and country origin^45^.

The studied EOs demonstrated significant insecticidal activity. The observed insecticidal activity and the differences observed in the efficiencies of the tested EOs might be attributable to the different bioactive compounds present in the different plant species. Chemical compounds with a wide spectrum of insecticidal effects include phenols (1,8 cineole and carvacrol), alcohols (α-terpineol, terpinen-4-ol and linalool), aldehydes and ketones (camphor and citronellal), and monoterpene hydrocarbons (Camphene, α-pinene and *p*-cimene)^43,46–49^. In a related study, *α*-pinene and *β*-pinene presented high insecticidal activity against *T. castaneum*^50,51^, whereas carvacrol, thymol, and p-cymene were more active against *T. castaneum*^46^. In a study conducted by Yildirim et al.^47^, twenty-eight monoterpenes, including monoterpene hydrocarbons and oxygenated monoterpenes, were found to display significant insecticidal effects against *S. zeamais*.

The tested EOs in our study are likely rich sources of these components. Benchabane et al.^41^ have studied the insecticidal activity of *T. pallescens* EO and reported that thymol and carvacrol were the components most responsible for this bioactivity. In this study, geraniol (20.86%), limonene (10.50%), and camphene (7.80%) were found to be the most abundant components in *C. citratus* EO. These compounds have also been reported to have insecticidal activities against a variety of pest insect species^50,52,53^.

Oyedejiet al.^54^ reported fumigant toxicity and contact activity of *Citrus sinensis* essential oil, and their constituents (including terpineol and linalool) against *S. zeamais*, with LC_50_ of 80.01 μL/L air and 95.63 μg/, respectively. Aryal et al.^55^ showed the stronger contact toxicity of *Acorus calamus* with LD_50_ value of 2.29 and 0.16% of EO concentration in scintillating vial bioassay to Maize weevil (*S. zeamais*). In the same technique, at 10% concentration *S. zeamais* adult showed highest repellent activity (98.75%) as compared to 5, 2.5 and 1.25% concentrations after 24 h of treatment. EO from inflorescences of *Etlingera elatior* had fumigant activity (LC_50_ = 0.83 μL/L of air) and contact activity (LD_50_ = 1.42 μg/adult) against adult forms of *S. zeamais*^56^. The EO from *Lippia javanica* (Burm. f.) showed fumigant toxicity to *S. zeamais* adult, with LD_50_ = 216 μg/cm3 air. This same EO also exhibited contact toxicity (LC50 = 6.22 mg/mL) and its major constituents (perillaldehyde (LC_50_ = 1.07 mg/mL) and linalool (LC_50_ = 1.82 mg/mL)) as well as a 1:1 mixture of these compounds (LC_50_ = 0.85 mg/mL), the mixture of the major compounds proved the most efficient^57^. The EO from *Croton rudolphianus* leaves exhibited toxicity against *S. Zeamais* by contact (LC_50=_70.64 μL/mL) and fumigation (64 μL/L in the air) caused the highest mortality (43.75%)^58^. Fouad et al.^59^ reported insecticidal activity of five EO (*Citrus aurantifolia* and *Citrus reticulate, Eucalyptus citriodora*, *Melaleuca alternifolia* and *Mentha piperita*), and their constituents (including Limonene, α-pinene and -α-pinene) against *S. zeamais*. The mean lethal concentrations (LC_50_) estimated for the EOs is variable between 1.04 and 2.56 μL g^–1^. Compared with 0.046 μL g^–1^ prorogued with deltamethrin-piperonyl butoxide insecticide. The contact and fumigant toxicity of EO against *C. maculatus* was comparable to *Rosmarinus officinalis* EO^60^, greater than *Petroselinum sativum* EO^61^ and less than *Thymus persicus* EO^62^.

Zhang et al.^63^ found that EO extracted from the leaves of *Chamaecyparis obtusa* demonstrated contact and Fumigant toxicity against *T.castaneum* adults, with respective LC_50_ values was 52.54 μg/adult of 7.09 μg/L air. In previous studies, it had been reported that the EO of *Artemisia herba alba Asso*, *Juniperus phoenicea* L and *Rosmarinus officinalis* exerted significant fumigant toxicity against *T. castanum* (LD_50_ = 12.4 μg/adult)^64^. LC_50_ values of EOs isolated from *Curcuma aromatica* salisb showed more contact (at 24 h, LC_50_ = 10.40 mg/cm2) and fumigant (at 24 h, LC_50_ = 18.18 mg/L air) toxicity against *T. castaneum*^65^. The contact toxicity studied with *Cinnamomum glanduliferum* (Wall.) leaf EO showed contact effect against *T. castaneum*, indicating a low LD_50_/LC_50_ value (12.13 μg/adult and 104.67 μg/cm2, respectively)^66^. The fumigant studies with *C. austroindica* and *C. aromatica* EOs showed higher toxicity against *T. castaneum* at the exposure time of 24 h (LC_50=_ 35.65 & 29.10 μL/L), respectively^67^. Pervious study reported better contact activity for *Thymus capitatus* and *Origanum compactum* against *T. castaneum*. In the contact toxicity, LC_50_ values were 0.58 and 0.35 μL/cm2 after 24 h of exposure, respectively^68^.

The toxicity of *T. pallescens* and *C. citratus* EOs against two stored-product pests, *S. zeamais* and *T. castaneum,* were determined. The CM% results were found to vary with the type and concentration of the tested EOs and the insect species. *Thymus pallescens* EO demonstrated high toxicity compared to *C. citratus* EO. Adults of *S. zeamais* were more susceptible to both plant EOs than *T. castaneum*. The different responses observed between the two insect species could be attributed to morphological and behavioral differences. It was found that the insecticidal activity of both EOs was highly influenced by the tested toxicity method. Our results are in accordance with previous findings regarding insecticidal activities of various EOs and their major components against *S. zeamais* and *T. castaneum*^34,46,58,69,70^.

Furthermore, the obtained results indicated that LC_50_ values increased in the combined populations compared with the separate populations. The observed increase in LC_50_ values for both pest insects could be due to the increase in the total number of individuals. In addition, the synchronous presence of multiple primary pest species in a mixed-species population can affect control measures, particularly for targeted species^11^.

In fact, EO toxicity can be attributed to different mechanisms of action against the physiological processes in insect pests. Previous studies have shown that natural compounds can cause symptoms associated with neurotoxic activity, such as hyperactivity, seizures, and tremors, often followed by paralysis and death^71^. EOs and their major components also reported to result in the strong inhibition of AChE in congeneric insect pest species, such as *S. oryzae*^13^. This could explain the toxicity effects of *T. pallescens* and *C. citratus* EOs against *S. zeamais* and *T. castaneum*. The bioactive properties of EOs can vary depending on the molecules contained in the EOs, the insect species, the toxicity test used, and the dose used^72^.

Plant-derived EOs affect insect metabolism and development through various biochemical and physiological processes^26,73^. In this study, significant decreases in the levels of proteins and carbohydrates, and a significant increase in lipids have been recorded. The decreased protein contents observed for both pest insects may be associated with several factors, including a decrease in protein synthesis or an increase in protein breakdown to detoxify the bioactive molecules of EOs^74,75^. Yazdani et al.^75^ obtained similar results using EOs from *Thymus vulgaris* L. and *Origanum vulgare* L. against the hemolymph protein of lesser mulberry pyralid *Glyphodes pyloalis* Walker.

The carbohydrate contents significantly decreased following EO treatments in the tested pest insects. Similar results were obtained by Yazdani et al.^76^ in *G. pyloalis* following treatment with *T. vulgaris* and *O. vulgare* EOs and by Tarigan et al.^77^ in *T. castaneum* and *Callosobruchus maculatus* (F.) (Coleoptera: Chrysomelidae) treated with cardamom, cinnamon, and nutmeg EOs*.*

Insects typically convert carbohydrates into lipids and glycogens^37^, which could explain the decrease in carbohydrate levels and the increased lipid levels observed in the treated insects. The increase in lipid levels can also be explained by the establishment of insect teguments to prevent the harmful effects of EOs. According to Morgan^78^, the external covers of insects consist of an impermeable lipid layer, which typically contains alkanes, methyl branched alkanes, and alkenes. The observed increase in the lipid levels of the insect body may represent the activation of resistance mechanisms to counteract the stress of EOs. The obtained results agree also with the observations of Bouzar Essaïdi et al.^79^, who found that treatments with extracts from *Lantana camara* L. significantly enhanced lipid contents of the pine processionary moth *Thaumetopea pytiocampa*.

## Conclusion

EOs provide an effective and ecologically sustainable solution for the control of insect pests. Our study demonstrated that *T. pallescens* and *C. citratus* EOs were effective against *S. zeamais* and *T. castaneum* and could be used as an alternative and “safe” control method for reducing the negative economic impacts associated with these insect pests, as part of an integrated pest management strategy for stored products. Our results also revealed that the synchronized occurrence of *S. zeamais* and *T. castaneum* reduced EO efficiencies and, consequently, increased the LC_50_ values.

## Data Availability

The data for this study is available from the second corresponding author upon reasonable request.
